# Studies in the Psychology of Sex

**Published:** 1901-02

**Authors:** 


STUDIES IN THE PSYCHOLOGY OF SEX. The Evolution of Modesty. The Phenomena of Sexual Periodicity. Auto-Erotism. By HAVELOCK ELLIS. Pages xii.—275. Philadelphia: F. A. Davis Company. 1900. [Price, $2.00.]

Those who have followed Havelock Ellis in his studies on criminology, psychopathia sexualis, and allied subjects, will be pleased to know that the distinguished author has put forth a work of incomparable interest and value, and one most timely in this age of erotism and commercialism. The author is well qualified to write on the psychology of sex, and his opinion, and conclusions are weighty and convincing. In the present work, he deals chiefly with the evolution of modesty, the phenomena of sexual periodicity, autoerotism and several appendices, which treat of the influence of menstruation on the position of women, sexual periodicity in men and the autoerotic factor in religion.

The author deals with the subject in a masterly manner, giving copious quotations in support of his views upon the several theories advanced.

The relation of modesty to sexual life is pointed out in a very interesting manner, the author believing that, like all the closely allied emotions, it is based on fear—an agglomeration of fears, two especially, one of much easier than human origin and supplied solely by the female; the other of more distinctly human character, and of social, rather than sexual origin. The author then cites examples, showing the periodicity of modesty and lack of modesty in the lower animals and in the nude and semi-nude races of men. A large amount of observation and collected facts are brought together in this volume on subjects not taught in the schools and colleges or referred to in the text-books on medicine, and to the enquiring mind of the professional man it is a welcome addition to our otherwise scanty literature on this subject.

The book is well printed on good paper and strongly bound.

